# Reflectance confocal microscopy of multiple idiopathic eruptive macular pigmentation: A case report

**DOI:** 10.1111/srt.13110

**Published:** 2021-11-09

**Authors:** Yuan Zhu, Wenzhong Xiang

**Affiliations:** ^1^ Department of Dermatology, Hangzhou Third People's Hospital Zhejiang Chinese Medical University Hangzhou China; ^2^ Department of Dermatology, Hangzhou Third People's Hospital Affiliated Hangzhou Dermatology Hospital of Zhejiang University School of Medicine Hangzhou China

Idiopathic eruptive macular pigmentation (IEMP) is a rare condition which was described in 1978 by French doctors Degos et al.^1^ The first reference in the English literature was in 1996; Sanz de Galdeano et al.^2^ summarized the diagnostic criteria after collecting and observing five cases: (1) eruption of brownish, nonconfluent, asymptomatic macules involving the trunk, neck, and proximal extremities in children or adolescents; (2) absence of preceding inflammatory lesions; (3) no prior drug exposure; (4) basal cell layer hyperpigmentation of the epidermis and prominent dermal melanophages without visible basal layer damage or lichenoid inflammatory infiltrate; and (5) normal mast cell count.

The pathogenesis of IEMP remains unclear. It seems that sunlight is not important, as most lesions occur in photoprotected areas.^3^ And there have been no reported cases of IEMP with a family history.^4^ Hormonal factors may be involved in increased pigment production, as most patients are children or young adults.^5^ Other related factors like inflammatory stimuli, autoimmune phenomenon have been hypothesized.^6^, ^7^, ^8^ IEMP was considered to be self‐limited in months to years,^4^, ^9^ although one case was reported to persist for 21 years.^8^

Currently this rare disease has not been characterized with reflectance confocal microscopy (RCM), which has proven to be an important noninvasive diagnostic tool, especially to characterizing melanocytic‐related skin lesions.^10^ We report a case of multiple IEMP of a teenage girl, with a description of RCM images, and compared RCM images with histopathology.

A 12‐year‐old girl presented discrete class elliptic brown macules and patches on the neck, trunk, upper arms, and upper legs of more than 2 years’ duration, without any clinical symptoms, there was no previous history of an inflammatory process, erythema, scaling. The patient has no other underlying disease. She had no history of any specific food or drug medication, no history of exposure to radioactive or other toxic substances. Treatments for the skin condition prescribed by outside dermatologists included black light (PUVA) treatment, oral vitamin C and vitamin E, and external use hydroquinone cream. The local patches were slightly lightened, but not noticeable. Physical examination showed multiple, discrete, round to oval, brown macules and patches with a smooth surface were seen on the neck, trunk, upper arms, and upper legs; adjacent lesions have a tendency of fusion (Figure 1). Hair, nails, and mucosae were normal. Lesions failed to elicit Darier sign.

**FIGURE 1:**
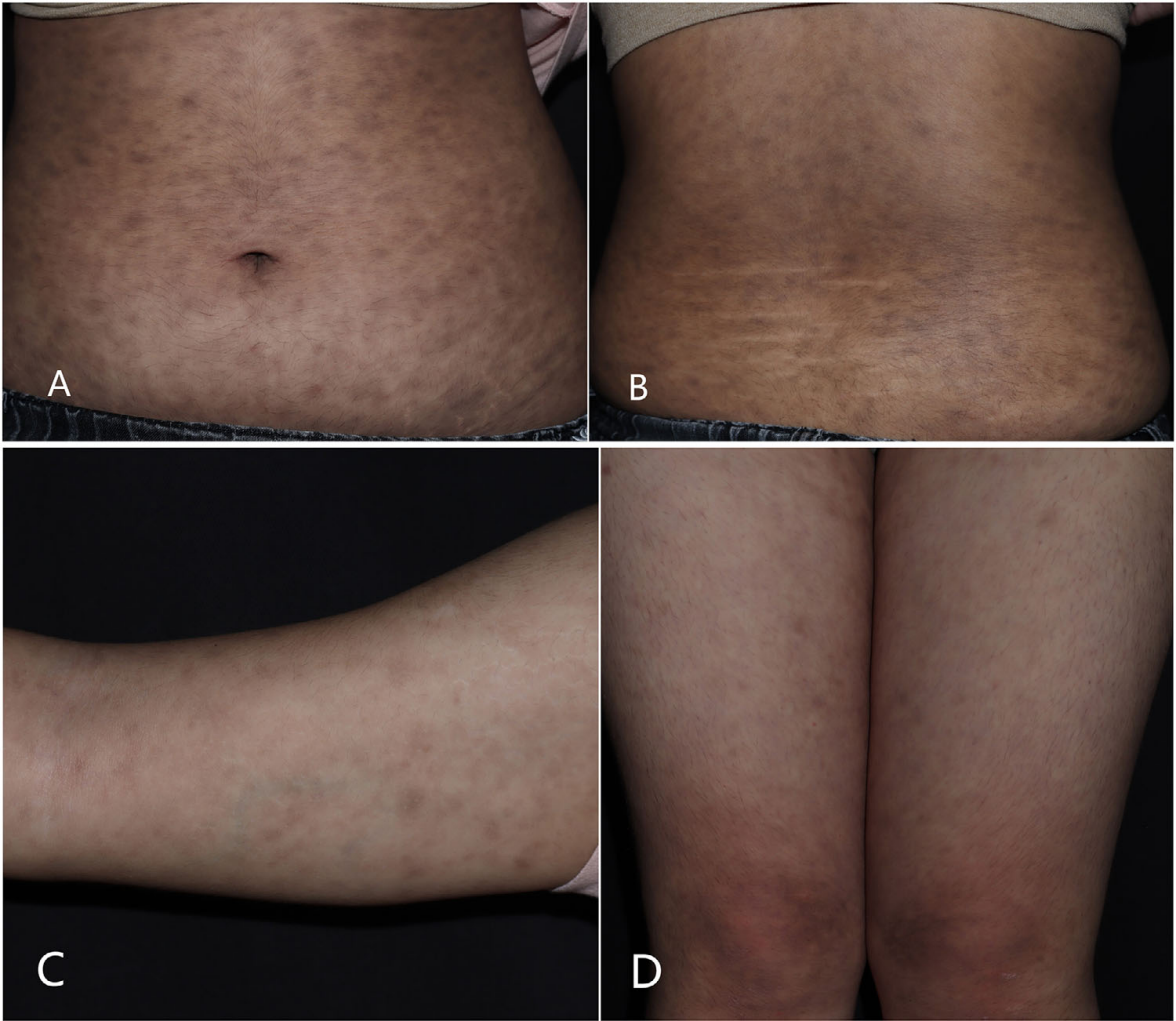
Multiple, brown, non‐scaly macules and patches. (A) Abdomen, (B) waist, (C) upper arm, (D) upper legs

Routine examination of the blood, urine, and stool and blood biochemistry were all in the normal range. RCM imaging revealed cuticle layer is normal, and the dermal papillary rings are intact. Compared with normal skin, the bright dermal papillary rings varying in size and shape were detected in the basal layer; hyper‐reflective melanophages can be seen in each of the dermal papillary rings. There are no melanocytes in the deep dermis below the dermal papillary layer (Figure 2). Histopathologic examination revealed the cuticle and spinous layers are normal, increased pigmentation in the basal‐cell layer, and scattered melanophages in the papillary dermis without visible basal‐cell layer damage or lichenoid inflammatory infiltrate and normal mast cell count. No abnormal pigment distribution was observed in the deeper dermis (Figure 3).^11^

**FIGURE 2:**
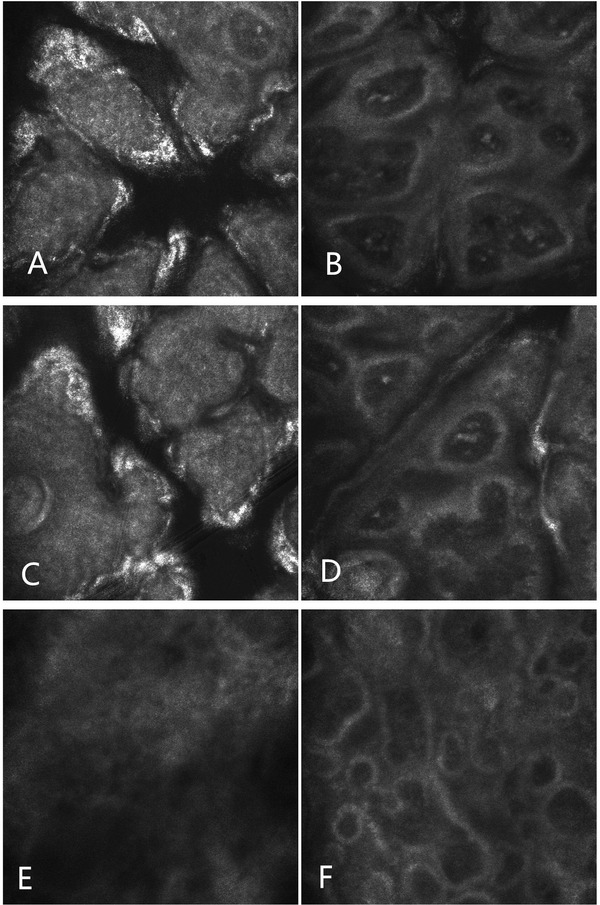
The reflectance confocal microscopy (RCM) images of the cuticle layer (A), basal layer (B), and dermis (E) of abdominal lesions, the cuticle (C) and basal layer (D) of lesions on the waist, the basal layer of the normal skin on the lower leg (F). The RCM images showed the cuticle layer of lesions is normal (A and C). Compared to the basal layer of normal skin in the lower leg (F), the dermal papillary rings are intact but with larger diameter, bright dermal papillary rings varying in size and shape were detected in the basal layer, hyper‐reflective melanophages can be seen in each of the dermal papillary rings (B and D). Melanocytes are not visible in the dermis below the superficial dermis (E). There was no obvious damage in basal layer or lichenoid inflammatory infiltrate of the three sites

**FIGURE 3:**
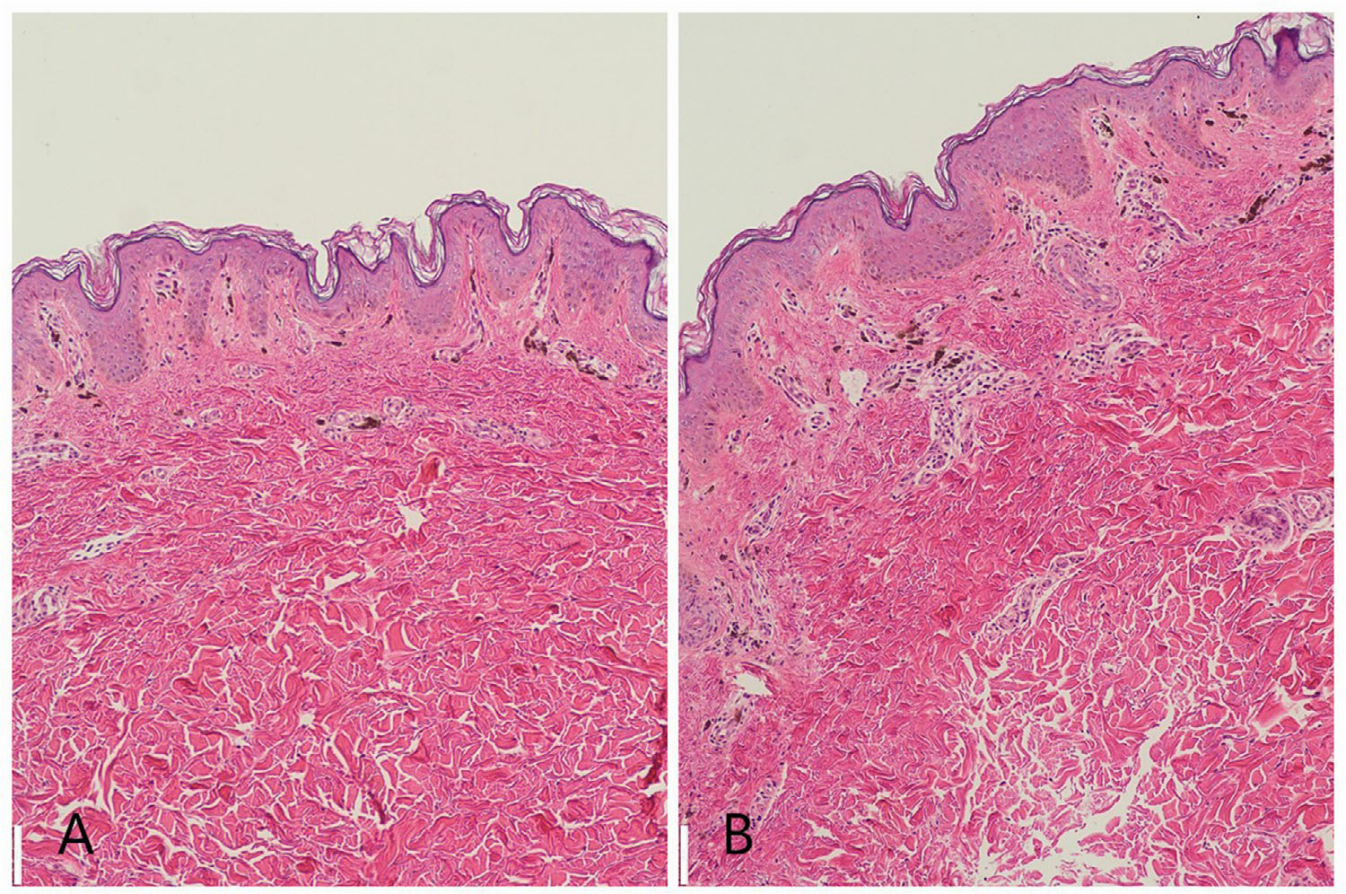
Histopathologic examination revealed the cuticle and spinous layers are normal, increased pigmentation of the basal‐cell layer, and scattered melanophages in each of the papillary dermis. No abnormal pigment distribution was observed in the dermis below the dermal papillary layer. No visible basal layer damage or lichenoid inflammatory infiltrate and normal mast cell counts. (Hematoxylin–eosin stain 4x original magnification)

All the findings in RCM images could be confirmed by histological examination. As far as we know, this is the first description of RCM features of IEMP. The description of this case is aimed to provide a better understanding of this rare disease. It is a supplement to the diagnostic criteria of such clinical pigmentation disease.

## References

[1] Degos R , Civatte J , Belaïch S . Idiopathic eruptive macular pigmentation (author's transl). Ann Dermatol Venereol. 1978;105(2):177–82. 677686

[2] Sanz de Galdeano C , Léauté‐Labrèze C , Bioulac‐Sage P , Nikolic M , Taïeb A . Idiopathic eruptive macular pigmentation: report of five patients. Pediatr Dermatol. 1996;13(4):274–7. 8844742 10.1111/j.1525-1470.1996.tb01237.x

[3] Câmara VM , Lupi O , Piñeiro‐Maceira J . Idiopathic eruptive macular pigmentation. Int J Dermatol. 2008 **;** 47(3):272–5. 18289331 10.1111/j.1365-4632.2008.03428.x

[4] Tsai WC , Lan J , Lee CH . Progression of idiopathic eruptive macular pigmentation in a girl from childhood to adolescence: case report and literature review. Pediatr Dermatol. 2016;33(5):e299–302. 27412039 10.1111/pde.12904

[5] Jang KA , Choi JH , Sung KS , Moon KC , Koh JK . Idiopathic eruptive macular pigmentation: report of 10 cases. J Am Acad Dermatol. 2001;44(2 Suppl):351–3. 11174413 10.1067/mjd.2001.103642

[6] Volz A , Metze D , Böhm M , Bruckner‐Tuderman L , Nashan D . Idiopathic eruptive macular pigmentation in a 7‐year‐old girl: case report and discussion of differences from erythema dyschromicum perstans. Br J Dermatol. 2007;157(4):839–40. 17714566 10.1111/j.1365-2133.2007.08127.x

[7] Milobratovic D , Djordjevic S , Vukicevic J , Bogdanovic Z . Idiopathic eruptive macular pigmentation associated with pregnancy and Hashimoto thyroiditis. J Am Acad Dermatol. 2005;52(5):919–21. 15858495 10.1016/j.jaad.2005.01.008

[8] Mehta S , Aasi S , Cole R , Chu P , Weinberg JM . Idiopathic eruptive macular pigmentation: a case of 21 years' duration. J Am Acad Dermatol. 2003;49(5 Suppl):280–2. 10.1016/s0190-9622(03)00745-x 14576654

[9] Wei‐Feng Z , Ai EX , Jun‐Fan C . Idiopathic eruptive macular pigmentation in a Chinese child. Indian Dermatol Online J. 2015;6(4):274–6. 26225334 10.4103/2229-5178.160266 PMC4513409

[10] Ruini C , Manfredini M , Pellacani G , Mandel VD , Tomasi A , Ponti G . Confocal microscopy characterization of BRAFV600E mutated melanomas. Melanoma Res. 2015;25(4):367–71. 26134486 10.1097/CMR.0000000000000147

[11] Kumarasinghe SPW , Pandya A , Chandran V , Rodrigues M , Dlova NC , Kang HY , et al. A global consensus statement on ashy dermatosis, erythema dyschromicum perstans, lichen planus pigmentosus, idiopathic eruptive macular pigmentation, and Riehl's melanosis. Int J Dermatol. 2019;58(3):263–72. 30176055 10.1111/ijd.14189

